# Gold (III) Derivatives in Colon Cancer Treatment

**DOI:** 10.3390/ijms23020724

**Published:** 2022-01-10

**Authors:** Agata Gurba, Przemysław Taciak, Mariusz Sacharczuk, Izabela Młynarczuk-Biały, Magdalena Bujalska-Zadrożny, Jakub Fichna

**Affiliations:** 1 Department of Pharmacodynamics, Faculty of Pharmacy, Medical University of Warsaw, 02-097 Warsaw, Poland; przemyslaw.taciak@wum.edu.pl (P.T.); mariusz.sacharczuk@wum.edu.pl (M.S.); magdalena.bujalska@wum.edu.pl (M.B.-Z.); 2 Department of Genomics, Institute of Genetics and Animal Breeding, Polish Academy of Sciences, Jastrzebiec, 05-552 Magdalenka, Poland; 3 Department for Histology and Embryology, Medical University of Warsaw, Chalubinskiego 5, 02-004 Warsaw, Poland; imlynarczuk@wum.edu.pl; 4 Department of Biochemistry, Faculty of Medicine, Medical University of Lodz, 92-215 Lodz, Poland; jakub.fichna@umed.lodz.pl

**Keywords:** gold, Au(III) complex, colorectal cancer, anticancer drugs, organometallic, cancer therapy, cytotoxicity, metallodrugs

## Abstract

Cancer is one of the leading causes of morbidity and mortality worldwide. Colorectal cancer (CRC) is the third most frequently diagnosed cancer in men and the second in women. Standard patterns of antitumor therapy, including cisplatin, are ineffective due to their lack of specificity for tumor cells, development of drug resistance, and severe side effects. For this reason, new methods and strategies for CRC treatment are urgently needed. Current research includes novel platinum (Pt)- and other metal-based drugs such as gold (Au), silver (Ag), iridium (Ir), or ruthenium (Ru). Au(III) compounds are promising drug candidates for CRC treatment due to their structural similarity to Pt(II). Their advantage is their relatively good solubility in water, but their disadvantage is an unsatisfactory stability under physiological conditions. Due to these limitations, work is still underway to improve the formula of Au(III) complexes by combining with various types of ligands capable of stabilizing the Au(III) cation and preventing its reduction under physiological conditions. This review summarizes the achievements in the field of stable Au(III) complexes with potential cytotoxic activity restricted to cancer cells. Moreover, it has been shown that not nucleic acids but various protein structures such as thioredoxin reductase (TrxR) mediate the antitumor effects of Au derivatives. The state of the art of the in vivo studies so far conducted is also described.

## 1. Introduction

Cancer is one of the leading causes of morbidity and mortality in the world, being responsible for approximately 9.6 million deaths in 2018 [1] and almost 10.0 million in 2020 [2]. According to global epidemiological data, colorectal cancer (CRC) is the third most frequently diagnosed cancer in men and the second in women. The worldwide burden of the disease is estimated to rise by 60% to over 2.2 million new cases and 1.1 million deaths by 2030. As reported in National Registry of Cancers, there has been a continuous increase in colon cancer incidence rate in Poland, with 4720 newly diagnosed cases [3,4].

Colon cancer develops as a result of the change of normal colonic epithelium including dysplasia and metaplasia to cancerous tumor, both polyposis and nonpolyposis, as a result of genetic alterations and the functional impact of these changes [5]. Colorectal tumors are driven by a wide range of spontaneous or induced mutations by mutagens, which is why they constitute a very heterogeneous group of cancers that are difficult to treat [6,7]. In the natural history of CRC development, a sequence of mutations accumulates, including antioncogene APC that becomes inactive and drives uncontrolled cell divisions. Further, K-Ras hyperactivity quickens cell divisions, and finally p53 becomes inactive. The accumulation of mutations in nonhereditary forms of CRC lasts about three decades [8]. 

It has been observed that median age at diagnosis with invasive cancer is about 70 years in developed countries [9]. The relationship between the aging and cancer is linked to the aging of lymphocytes (immunosenescence) [10] and with DNA defects that accumulate with age as well as with hormonal changes [11]. The development of CRC in a large number of cases begins decades before its detection through the adenoma–carcinoma sequence. By that time, it reaches a late stage, which complicates the treatment [12]. The risk for developing CRC is associated with personal features or habits [13] such as age [14], chronic diseases history including inflammatory bowel disease [15], Crohn’s disease [16] and sedentary lifestyle, obesity [17], unhealthy nutritional habits [18], smoking and alcohol consumption [19]. Therefore, a continuous increase in the incidence of CRC in developed countries can be attributed to an increasingly aging population, unfavorable modern eating habits and an increase in risk factors such as smoking, low physical activity and obesity [20]. 

In the case of early diagnosis, the basic treatment is surgery, but this is already ineffective in advanced cases with metastases, which constitute about 25% of diagnoses [21,22]. The effectiveness of a standard neoadjuvant cytotoxic therapy in these patients, based on oxaliplatin and other cisplatin analogues, has been drastically reduced by the lack of specificity towards cancer cells, rapid development of drug resistance and cancer recurrence [23]. 

Thus, current research has been focused on developing new metallodrugs based on other nonplatinum transition metals, such as gold (Au), silver (Ag), iridium (Ir) or ruthenium (Ru) [24]. Another approach is the substitution of a ligand and the modification of existing chemical structures that led to the synthesis of a wide range of metal-based compounds, some of which have shown improved cytotoxic and pharmacokinetic profiles [25]. Furthermore, nanoparticles, through their enhanced permeability and retention (EPR) effect, preferentially accumulate in tumors [26], which also makes them an attractive research topic. Despite an abundant literature on gold nanoparticles in experimental cancer biology, only a few of the gold-based nanodevices are currently being tested in clinical trials [27], and none of them are approved by health agencies. In recent years, the field of cytotoxicity of Au complexes has been rapidly developing, which is reflected in numerous reviews. Au(III) compounds seem to be a particularly promising and good alternative for platinum-based anticancer drugs due to their structural similarity to platinum (Pt)(II) [28]. However, most of the described compounds have an unclear mode of action and a lack of clinical relevance. That is why we are constantly looking for new and fully characterized Au(III) derivatives. In this review, we summarize the previous work and describe the most recent advances in the use of Au(III) derivatives in CRC treatment.

### 1.1. Colorectal Cancer Treatment

CRC can be divided into five stages: 0, I, II, III and IV, depending on the involvement of lymph nodes, formation of metastases and the grade of local invasion depth. The most advanced stage with the worst prognosis is stage IV. The therapeutic approach depends on the stage of the disease. Thus, tumors at stage 0 are surgically removed while patients with stage II and above (invasive cancer crossing the basement membrane) require more complex treatment methods that include surgery, chemotherapy and/or radiotherapy [12,29,30].

The standard therapy for CRC currently involves various medicines, either in combination or as single agents, such as: 5-fluorouracyl/leucovorin (5-FU/LV), capecitabine, irinotecan, oxaliplatin, bevacizumab, cetuximab, panitumumab, ziv-aflibercept, ramucirumab, regorafenib, trifluridine-tipiracil, pembrolizumab and nivolumab. The described mechanisms of action of cytostatics are varied and include interference in DNA replication and inhibition of the activities of vascular endothelial growth factor (VEGF) and epidermal growth factors (EGF) [31,32,33,34]. 

In surgical treatment of CRC that crosses the basement membrane, adjuvant and neoadjuvant therapy is proposed to be the gold standard. These terms (adjuvant and neoadjuvant) refer to the pairing of sequential steps of therapy, that is, chemotherapy followed by surgery, or surgery followed by chemotherapy, respectively.

In context of CRC therapy, the clinical prospective data on neoadjuvat therapy is limited—patients can benefit from these treatments [35], however the limitations are side effects such as neuropathy induced by oxaliplatin—that force for the invention of better and safer alternatives, such as other metallodrugs, including gold complexes [29].

The main reason for the low effectiveness of CRC treatment is the limited bioavailability and the lack of specificity towards cancer cells of conventional chemotherapeutic agents, which contributes to the destruction not only of cancerous cells but also of normal cells, and consequently leads to serious side effects. In addition, the development of drug resistance is observed [26,36,37,38,39].

In order to optimize anticancer therapy and reduce side effects, new alternative therapies in CRC are being investigated [13]. The most promising trends of research are the use of agarose tumor macrobeads [40,41,42], anti-inflammatory drugs [43,44,45], probiotics [46,47,48] and metal-based drugs [29,49,50,51,52].

### 1.2. Metallodrugs

Laboratories and scientific institutions around the world have researched many derivatives of metals such as Pt, Au and other metals for the treatment of tumors. Moreover, some of them have been patented or even implemented. A well-known drug is cisplatin, whose potent anticancer effect is derived from the interaction with DNA and impaired processes of replication, transcription and translation [53,54]. Unfortunately, the use of cisplatin may lead to neuro- and nephron-toxicity, along with evidence of either induced or intrinsic resistance to the treatment in some tumors [55,56]. Thus, development of both novel Pt- and other metal-based compounds is needed in order to obtain complexes with higher effectiveness, increased selectivity for tumor tissue, reduced toxicity, wider spectrum of activity, and ability to overcome tumor resistance often arising from cisplatin treatment [57].

Currently, the most abundant group of non-Pt, metal-based anticancer preparations are those incorporating Au. In fact, the group of reported compounds exhibiting anticancer activity based on Pt is less numerous than the group with same activity based on Au. Further down, medicinal preparations based on metallic Au (Au 0), also known as—depending on the size of particles—colloidal or nanoparticle Au, are by far the largest group among all therapeutic Au-based compounds [39,58,59].

Metallic Au particles are insoluble in blood and plasma, but the tumor cells exhibit the ability to greater accumulation of Au particles in relation to normal cells according to the EPR effect [60,61]. Despite the promising results of preclinical studies, low bioavailability and very rapid excretion from the body discourage further attempts to use metallic Au in the clinical setting [62,63]. Moreover, indicated accumulation of such compounds of Au in the liver and spleen is an important side effect [64,65,66,67].

The low bioavailability of Au(0)-based drugs has initiated research into Au complexes that are characterized by better bioavailability and solubility. During the last two decades, a large variety of Au(I) and Au(III) compounds are reported to possess relevant antiproliferative properties in vitro against selected human tumor cell lines, qualifying them as excellent candidates for further pharmacological evaluation. The unique chemical properties of the Au center confer very interesting and innovative pharmacological profiles to Au-based metallodrugs [68]. As previously described, Au(I) and Au(III) compounds are widely used in research as anticancer agents, however, most of them display limitations concerning solution stability under physiological conditions [24,25,29,69,70,71,72,73,74,75,76]. Au(III) compounds seem to be particularly promising and a good alternative for Pt-based anticancer drugs, due to their structural similarity [28]. Currently, researchers aim to develop different types of ligands able to stabilize the Au(III) cation and prevent its reduction under physiological conditions.

### 1.3. Perspectives for Gold-Based Compounds against Colorectal Cancer

There are several properties of Au that make it a potential anticancer agent in CRC. Since Au compounds have been used for centuries in the treatment of rheumatoid arthritis; their well-known anti-inflammatory and immunosuppressing properties made them promising drug candidates for CRC treatment [77]. 

Moreover, there is evidence that not nucleic acids, as in the case of cisplatin, but some selected protein targets, for example, thioredoxin reductase (TrxR), mediate the antitumor effects of Au derivatives [29,78,79,80]. This is particularly important as Trx-1 expression is upregulated in several human cancers, including CRC. The Trx/TrxR redox pathway is an attractive target for the development of new anticancer drugs, as elevated Trx-1 levels result in rapid tumor growth, inhibition of apoptosis, and reduced patient survival [81,82]. Gold complexes have multimodal mechanisms of action, and examples of them are listed below. 

Direct DNA interaction—platin-derived compounds such as oxaliplatin are standard DNA-binding therapeutics applied in CRC—it was shown that some gold complexes cause DNA fragmentation rather than cross-linkage as a result of reversible and noncovalent DNA interaction [83,84,85,86].

Gold complexes can induce apoptosis by intrinsic mechanisms involving caspase 9 and 3 activation, cytochrome C release, and PARP cleavage [80,87,88,89].

Protein kinase C (PKC) is involved in cell proliferation, differentiation, migration, and survival. Hyperactivation of PKC signaling can be observed in cancers including CRC. Some gold complexes including aurothioglucose and aurothiomalate can inhibit PKC and consequently inhibit proliferation of cancer cells [90,91]. 

MEK/ERK (Ras) pathway—this pathway is necessary for proper cell divisions, and if hyperactivated it drives unhampered divisions of the cancer cell, its motility, mobility and insensitivity to induction of apoptosis. Ras hyperactivation is often involved in CRC development, but all signaling elements including growth receptors and downstream kinases (Raf, MEK, ERK) might participate in cancer progression [92].

The proteasome–ubiquitin pathway (UPS)—this cellular protein destination and degradation system was shown to be a good anticancer drug target, since proteasome inhibitors are applied in cancer treatment. Tumor cells with deregulated cell divisions are more sensitive to inhibition of UPS than normal cells that can enter the cell cycle, and block and escape death by proteasome inhibition. Particular gold complexes such as the Au(III) dithiocarbamate compound turned out to inhibit proteasome activity and induce accumulation of polyubiquitin complexes, both in vitro in tumor cell lines, as well as in xenografts resected from experimental animals [83,93].

Thus, the advantage of Au complexes is their multimodal mechanism of action targeting various elements crucial for cancer progression.

## 2. In Vitro Studies

Au(III) compounds, due to their similarity to cisplatin, were among the first metal complexes tested for anticancer activity. It was initially assumed that the mechanism of action of these compounds would be the same as Pt-based drugs, and based mainly on interactions with DNA [94,95]. However, subsequent studies revealed that the cytostatic effect of Au derivatives is rather multifaceted, and may include some selected proteins such as TrxR or deubiquitinases [78,79,80,96,97]. It is also known that these proteins are overexpressed in cancer cells, and inhibition of their activity is lethal to cancer cells, although with a much lower effect on noncancerous cells. Therefore, it is believed that the systemic toxicity of Au complexes will be significantly reduced compared to conventional therapy [29]. Notwithstanding, most of them display limitations concerning solution stability under physiological conditions and are easily reduced to Au(I) or Au(0), thus losing their activity. However, as the following examples show, it is possible to synthesize stable, resilient-to-reduction, organogold(III) complexes, which is the main goal in this field. To date, according to our knowledge, inorganic Au(III) complexes have not been described as cytostatic agents in CRC.

### 2.1. Organogold Derivatives

**Figure 1:**
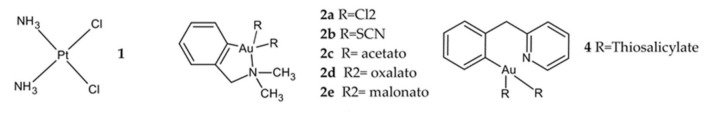
Structural comparison of organogold derivatives **2a**–**e**, **4** with cisplatin (**1**).

One of the first studies of Au(III) compounds on colon cancer cell lines was not satisfactory. In 1996, four analogues of the Au(III) complex [AuCl2(damp)] (damp = 2-[(dimethylamino)methyl]-phenyl) (**2a**–**e**) were evaluated for antitumor activity. The compounds have structural features in common with cisplatin, which was included as a comparison in the study (Figure 1). The derivatives have been tested on a panel of cell lines, among others made from human colorectal cancer such as: SW620, SW1116, SW403, HT29/219. The comparison of results for Au(III) compounds and cisplatin showed broadly similar growth-inhibiting properties and differential cytotoxicity, and the SW620 and SW1116 lines were the least sensitive to the compounds (Table 1). Therefore, these complexes might have the potential as an antitumor agent but in selected cancer types. In addition, although some compounds had some structural similarity to cisplatin, their mode of action seemed different [98]. Furthermore, the organogold compounds studied exhibit good stability within a physiological-like environment. Subsequent chemical and biological studies of **2a**–**e** derivatives also confirmed the same properties [99].

**Figure 2:**
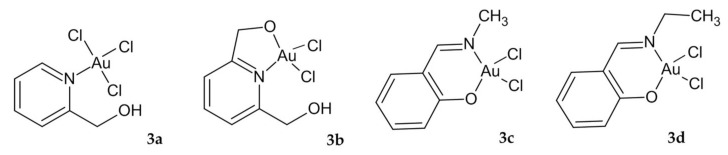
Au complexes **3a**–**d** with a square-planar geometry.

Calamai et al. designed, synthesized and evaluated for cytotoxicity four complexes (**3a**–**3d**, Figure 2) with a square-planar geometry, like cisplatin. The experiment was performed on a panel of five tumor cell lines, composed mainly of cell lines sensitive to cisplatin, e.g., HCT-8 with cisplatin and sodium tetrachloroaurate (NaAuCl_3_) as control. In a colon cancer cell line, all four investigated derivatives exhibited less cytotoxic effect (with IC_50_ ranging from 8 to 29 µΜ) than cisplatin (IC_50_ value 3.9 µΜ). On the contrary, their antitumor potency against other tumor cell lines was comparable to or even greater than cisplatin (Table 2) [84]. 

Cytostatic activity for square-planar cycloaurated Au(III) compounds on HCT-116 and HT29 cell lines was also studied. The most active thiosalicylate derivative **4** (Figure 1), with IC_50_ value 11.7 µM for HT29, was further tested in vivo. Additionally, it was proposed that the molecular targets of these compounds are thiol-containing biological molecules such as the cathepsin cysteine proteases, and it was found that they are able to inhibit both cathepsins B and K [100]. 

**Figure 3:**
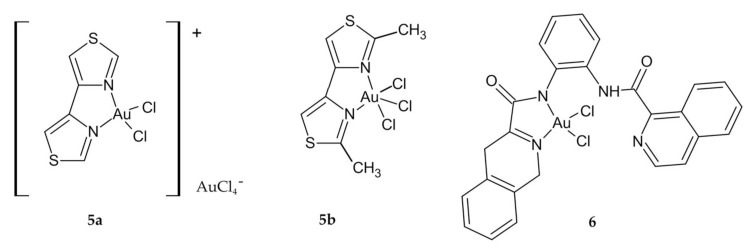
Au derivatives **5a**,**b** and **6**.

The cytotoxicity of two sterically different bithiazole Au(III) complexes, regular square-planar compound **5a** and disordered square-pyramidal geometry in **5b** (Figure 3), was also investigated. Of the three cancer cell lines studied, derivative **5a** showed no cytotoxicity in Caco-2, but its toxicity in HT29 was similar to cisplatin (Table 2). Compound **5b** had no anticancer value because it exhibited very little toxicity on the studied cell lines. This lack of activity might be due to steric construction [101].

Wilson et al. reported that out of a series of four square-planar Au(III) chelates, only isoquinolylamidogold(III) chelate **6** (Figure 3) was sufficiently cytotoxic in the single-dose assay and promising for further studies. The data indicate that seven different colon cell lines were among the most susceptible to the Au(III) complex, with IC_50_ values below 20 μM. The lowest IC_50_ value was for the colon cancer cell line SW-620 (Table 2). The cytotoxicity of the investigated compound compares favorably with that of cisplatin and etoposide (a nonintercalating topoisomerase II inhibitor). Dual topoisomerase I and II inhibitors were given as the mechanism of action of the compounds. However, there is a need to improve the structure of the chelates so that they are less susceptible to precipitation from aqueous solutions, which may increase their cytotoxicity and thus the chances of further development [85].

**Figure 4:**
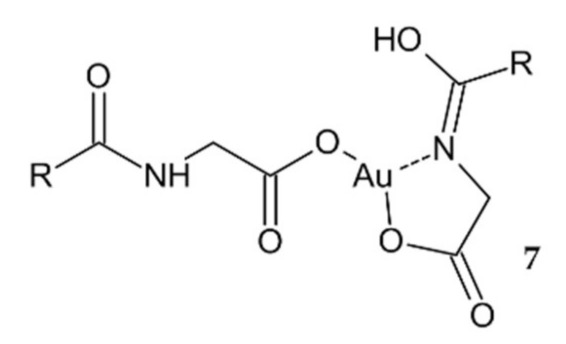
A new bile acid cholylglycinato Au(III) complex **7**.

Another approach was presented by researchers who synthesized a new bile acid cholylglycinato Au(III) complex **7** (Figure 4) based on the ability of bile acids for vectorializing the cytostatic activity of other agents. Cytostatic effect of the investigated compound was mild against human colon adenocarcinoma LS-174T in vitro, but **7** had a significantly higher IC_50_ value than cisplatin (Table 2). The appearance of colloidal Au during the process of hydrolysis under physiological conditions may explain the low cytostatic activity [102].

**Figure 5:**
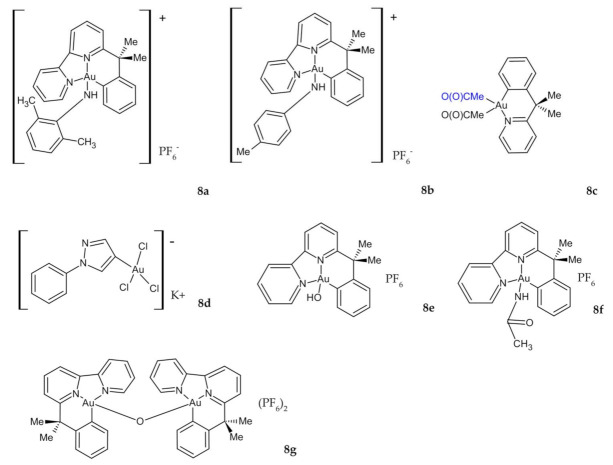
Novel organogold (III) compounds **8a**–**g**.

Investigations of the cytotoxicity scores of novel organogold (III) compounds **8a**–**d** (Figure 5) revealed that all of these compounds, except for **8d**, are generally stable under physiological conditions and exhibit significant cytotoxic properties on a limited panel of human tumor cell lines. However, negligible anticancer effects (Table 2) were generally measured on the HT29 line compared to those of cisplatin and oxaliplatin [103]. Massai et al. published further work on derivatives of these cyclometallated complexes **8e,g** (Figure 5). All three compounds, especially **8g**, cause moderate, but still significant. antiproliferative effects toward HCT-116 cancer cells, accompanied by a strong induction of apoptosis and a G0/G1 cell cycle arrest (Table 2). Given the fact that all these effects were greater on CRC cell line HCT-116 compared to the normal L-929 fibroblast cell line, there is a possibility to further investigate and characterize **8g** as a potential anticancer agent on a larger and more complex panel of cancer cell lines, as well as to evaluate the mechanisms of its different toxicity effects between in vitro models of tumoral and healthy tissue [104].

**Figure 6:**
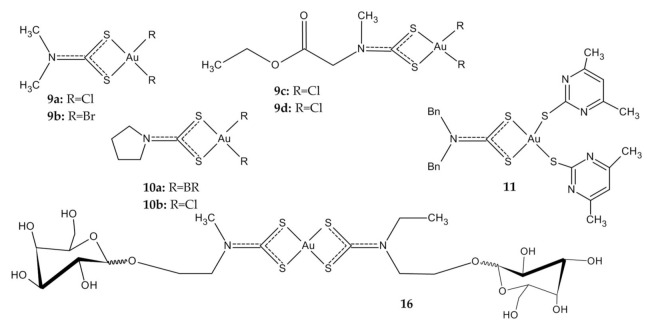
Au(III) dithiocarbamate derivatives **9a**–**d**, **10a**,**b**, **11**, **16**.

Considering the ability of dithiocarbamates to act as chelating ligands, many examples of Au(III) dithiocarbamate derivatives have been reported. The first more promising studies on the cytotoxic activity of Au(III) compounds in CRC were published by Ronconi et al., which described some of Au(I) and Au(III) complexes with dithiocarbamate ligands (DMDT = N,Ndimethyldithiocarbamate; DMDTM = S-methyl-N,N-dimethyldithiocarbamate; ESDT = thylsarcosinedithiocarbamate). Their preliminary studies have shown that the biological activity of the compounds should generally be attributed to the presence of the Au(III) metal center, and that the Au(I) compounds produce a less pronounced inhibition of cell growth compared to Au(III) analogues. Four complexes **9a**–**d** (Figure 6) were selected for further in vitro cytotoxicity testing. Data regarding their in vitro antiproliferative activity against colon adenocarcinoma cell lines (LoVo), which are notoriously not very sensitive to cisplatin, are extremely interesting, because these new Au(III) complexes seem to also be cytotoxic against tumor cell lines resistant to cisplatin, overcoming their intrinsic resistance and supporting the hypothesis of a different mechanism of action. The IC_50_ values of investigated compounds ranged from 2.4 nM to 7.9 µΜ (Table 2), while cisplatin’s IC_50_ value was 56 µΜ [105,106].

Treatment with the other two Au(III) compounds based on the pyrrolidinedithiocarbamates (PDT), **10a,b** (Figure 6), showed a rapid (three-hour) dose-dependent decrease in cell viability in the HCT-116 colorectal carcinoma cells. It was found that the bromide derivative **10a** was more effective than the chloride one **10b** in inducing cell death and acting via elicited oxidative stress, with effects on the permeability transition pore, a mitochondrial channel whose opening leads to cell death (Table 2). Cisplatin did not show any cytotoxicity under the same experimental conditions [107]. Greater cytotoxicity of bromides is in agreement with the findings reported by Casini et al. [108] for other Au(III) anticancer agents.

Mixed thiolate–dithiocarbamate Au(III) complexes display high antiproliferative activity against colon cancer cell line Caco-2/TC7, without affecting differentiated enterocytes. The most promising derivative **11** (Figure 6) is characterized by a much higher cytotoxicity compared to cisplatin, and slightly higher than auranorfin (Table 2). Although it was assumed that TrxR was a potential target of the dithiocarbamate Au complexes, this was not supported, as reactive oxygen species (ROS) levels and TrxR activity remained unchanged during the experiment. Cell death studies showed that the complexes induced changes in mitochondrial membrane potential, cytochrome C release and caspase-3 activation. The complexes are characterized by high stability under physiological conditions, which gives the opportunity to develop new cytostatics in the treatment of colorectal cancer with the proteasome as a possible target [88].

**Figure 7:**
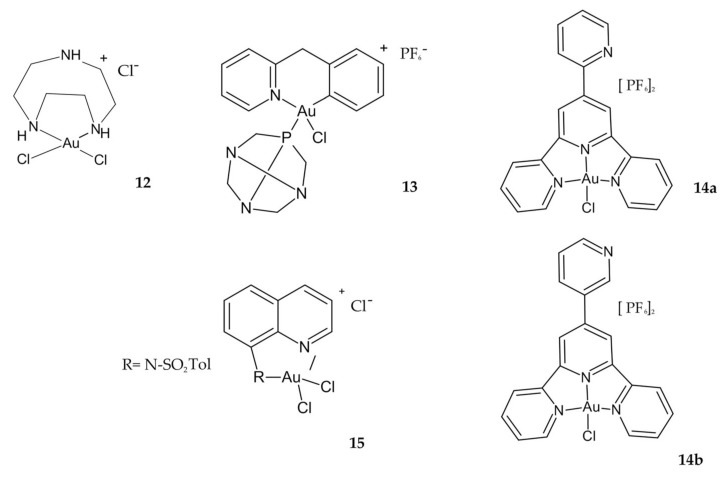
Au(III) complexes **12**, **13**, **14a,b**, **15**.

Another study showing high cytotoxicity against the colon cancer cell line was reported by Shi et al. Compared to cisplatin, Au(III) compound **12** (Figure 7) has demonstrated higher cytotoxicity for HCT-116 cell lines at all the concentrations used in the studies. At the concentration of 106 M, the compound showed 30% inhibition against the HCT-116 cell line, while at the same concentration, cisplatin shows 20% inhibition. Additionally, it has been shown that the compound **12** can induce DNA double helix distortion in its mechanism of action [86].

The new Au(III) cyclometallated phosphine derivative **13,** with PTA = 1,3,5-triazaphosphaadamantane ligand (Figure 7), which is not cytotoxic but is known in general to improve water solubility, has been proposed as a new antineoplastic agent in colorectal cancer. The derivative **13** was the most active of all investigated complexes in the study, twice as toxic as cisplatin against HCT-116 p53+/+ cells, and poorly effective on HCT-116 p53−/− (Table 2). This result suggests a similar dependence on p53 pathways for Au(III) complex as for cisplatin. Interestingly, compound **13** inhibited the zinc finger enzyme PARP-1 in nM concentrations, suggesting the possible design of selective inhibitors and the use of organometallic Au compounds in combination therapies with other anticancer drugs [89].

Both Au(III) complexes **14a** and **14b** incorporating 2,2′:6′,2′′-terpyridine ligand (Figure 7), showed excellent antiproliferative activities against HCT-116, higher than the free ligands and cisplatin. Additionally, compound **14a** showed high selectivity against HCT-116 and HCT-116p53 −/− cells, confirmed by the selectivity index (SI). Most interestingly, the complex **14a** exhibited proapoptotic activation, while **14b** displayed pronecrotic actions [109].

Au(III) complexes containing quinoline ligands at position 8 with different groups arose due to the broad spectrum of medical applications of 8-hydroxyquinoline. It was found that compound **15** with an N-tosyl-8-aminoquinoline ligand (Figure 7) is the most active of the synthesized complexes in all cancer cell lines tested, including the cisplatin-resistant WiDr cell line, and acts by interacting with proteins. Moreover, this complex has proved to be the most stable compound in DMSO and saline solution, even after several hours [110]. 

The latest available work in this area describes Au(III) complexes with glycoconjugated dithiocarbamato ligands (among others **16**, Figure 6). To improve the selective accumulation of an anticancer metal payload in malignant cells, carbohydrates (D-glucose, D-galactose, and D-mannose) were chosen as targeting agents exploiting the Warburg effect that accounts for the overexpression of glucose-transporter proteins (in particular GLUTs) in the phospholipid bilayer of most cancer cells. Unfortunately, the collected results indicate that the Au(III) complexes are not good substrates for GLUT and are inactive toward HCT-116 cells, with IC_50_ values higher than 50 µM (Table 2) [111].

### 2.2. Porphyrin Complexes

More comprehensive and promising results were presented by using porphyrin ligand, which can stabilize the Au(III) ion against demetallation and reduction by the biological reductant glutathione [112]. 

**Figure 8:**
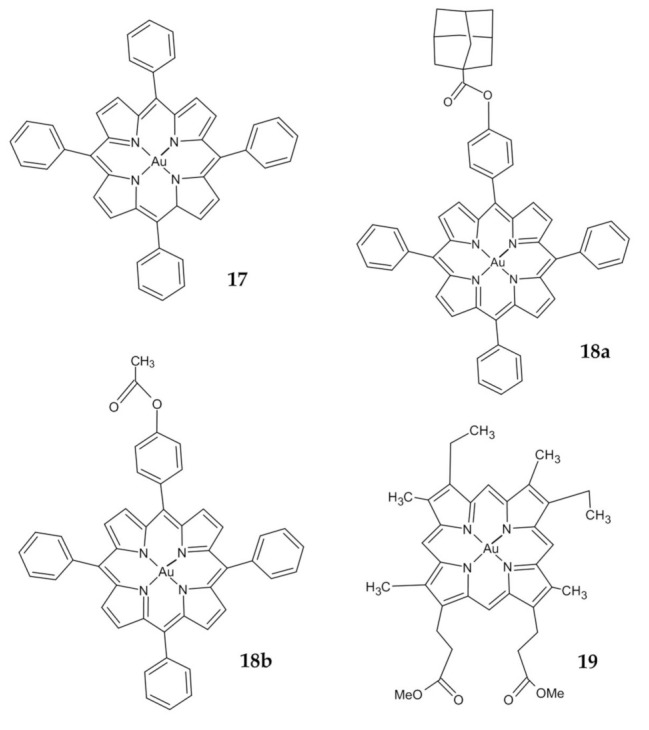
Au(III) porphyrin complexes **17**, **18a,b**, **19**.

Preliminary studies of a series of Au(III) tetraarylporphyrin (TPP) derivatives confirmed their stability in the presence of glutathione and demonstrated a much greater potency than cisplatin in killing human cancer cells, including drug-resistant variants [113]. The **17** complex (Figure 8) was selected for further study of its antitumor activity and its mechanism against colon cancer. The investigated compound exhibited marked cytotoxicity against different colon cancer cell lines and IC_50_ values with 9-fold to 21-fold greater potency than that of cisplatin (Table 3). Furthermore, the **17** complex significantly induced apoptosis and cell cycle arrest and cleaved caspase 3, caspase 7, and poly(ADP-ribose) polymerase; released cytochrome C, and upregulated p53, p21, p27, and Bax. Furthermore, in vivo tests were also carried out [114]. The same complex was further studied by Altaf et al. with promising results. Compound **17** showed very high activity compared to Au(III) complexes of meso-1,2-di(1-naphthyl)-1,2-diaminoethane and cisplatin. It was about 7–8 times more potent than cisplatin against the HCT-15 cancer cell [115]. 

A novel Au(III) porphyrin analogs **18a** and **18b** (Figure 8) were prepared by modifying one of the peripheral phenyl groups of **17**. Results revealed that **18b** was more cytotoxic to the colon cancer line than **18a** (Table 3). The investigated complexes reduced the survival of human CRC HT-29 and HCT-116 cell lines, caused cell cycle arrest in the G2/M phase, and decreased expression of cyclin B1 and cyclin-dependent kinase 1 (Cdk1) was observed with an increase in regulation of the active form of p53, p21, and Bcl-2 associated with X (Bax) [87]. Furthermore, they induced apoptosis by the intrinsic pathway, as previously described [114]. 

Tong et al. demonstrate an anticancer activity of Au(III) mesoporphyrin IX dimethyl ester (**19**) (Figure 8). This compound displayed a higher cytotoxicity in HCT-116 colon cancer cells compared to noncancerous colon epithelial cells (NCM460) with 25-fold differences in IC_50_ values (Table 3). Promising results from in vivo studies were also reported. The mechanism of action involves modification of the reactive cysteine residues and inhibiting the activity of thioredoxin, peroxiredoxin and deubiquitinases. Crucially, this study revealed that Au(III) induced ligand scaffold reactivity to target the thiol, which could be a useful tool in oncology [93].

### 2.3. N-Heterocyclic Carbenes (NHCs) Derivatives

A large part of the reviewed papers concerns research on N-heterocyclic carbenes (NHCs) Au complexes. It is widely accepted that the replacement of phosphine ligands by the isolobal NHC ligands frequently improves the properties of the new compounds for practical applications, which are them being water- and air-stable and easier to handle. Moreover, the imidazolium salts as the ligand precursor can be functionalized with almost any substituent, a rarely accessible feature in phosphines [116]. 

**Figure 9:**
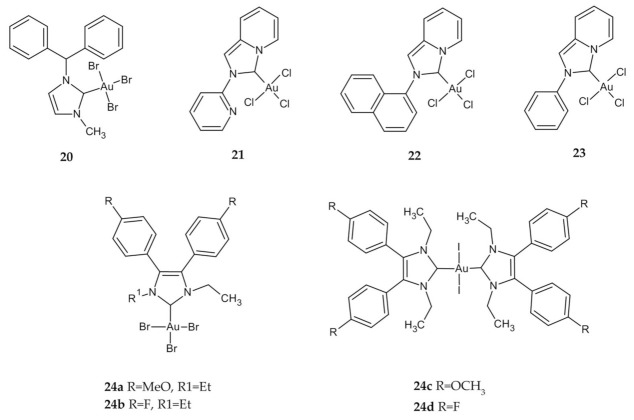
NHCs Au(III) derivatives **20**–**24**.

Lemke et al. described the synthesis and antiproliferative activity of a new halide, amino acid and dipeptide NHC Au(I) and NHC Au(III) complexes. In vitro cytostatic effect of compound **20** (Figure 9) was only mild against human colon adenocarcinoma HT-29, with an IC_50_ value higher than most active complexes and slightly higher compared to cisplatin (Table 4) [116].

Other groups were evaluated in vitro for the cytotoxicities of Au and Ag NHCs complexes supported by a pyridine, annulated imidazole-2-ylidene (**21**) [117,118], imidazolium salt 1-naphthyl-2-pyridin-2-yl-2H-imidazo[1,5-a]pyridin-4-ylium hexafluorophosphate (**22**) [119], pyridyl[1,2-a]{2-acetylylphenylimidazol}-3-ylidene (**23**) [120] (Figure 9). All complexes of Au(III)–NHC exhibited lower cytotoxicity than Ag(I) and Au(I) complexes for nearly all cell lines tested. Despite that, compounds **21**, **22**, and **23** were more potent than cisplatin against the HCT-116 cell line (Table 4). Unfortunately, due to the lower cytotoxicity of Au(III)–NHC, studies involving the mechanism of action have not been performed for this complex.

Liu et al. have proposed NHC–Au halide complexes derived from 4,5-diarylimidazoles. The influence of the oxidation state of the metal (Au(I) or Au(III)) is relatively low in general, however, on the HT-29 cell line, the Au(III) complexes **24a** and **24b** (Figure 9) were less active than their Au(I) congeners, which coincides with earlier papers. All complexes inhibit TrxR with different IC_50_ values, nevertheless, other targets should be considered as part of the mode of action [121]. Researchers, encouraged by these promising results, have investigated the effect of halide exchange in Au–NHC complexes on their pharmacological properties. The growth-inhibitory effect against HT-29 cells was more than 10-fold higher for complexes **24c** and **24d** (Figure 9) than that of cisplatin or 5-FU. Moreover, this effect was independent of the oxidation state of Au and type of halides. Although the investigated complexes were successfully accumulated in the neoplastic tissue and localized in large amounts in the cell nucleus, their mode of action has not been clearly defined [122].

**Figure 10:**
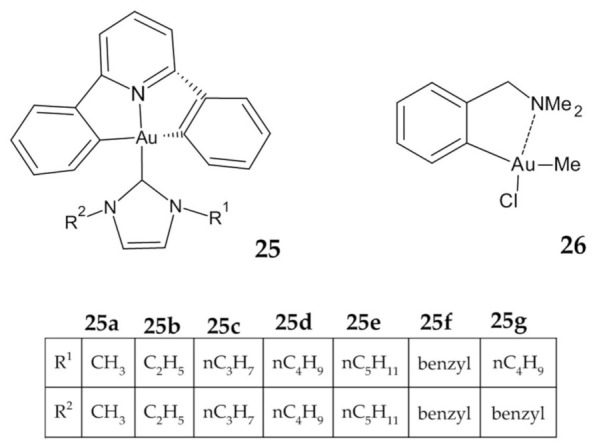
Cyclometalated Au(III) complexes **25**, **26**.

Fung et al. described the identification of multiple molecular targets for cyclometalated Au(III) complexes containing NHC ligands using photoaffinity groups. The IC_50_ values for complexes **25a**–**g** (Figure 10) ranged from 4.4 µM to 0.2 µM in comparison to cisplatin with an IC_50_ value 11.8 µM (Table 4). Importantly, all the compounds showed much higher cytotoxicity to HCT-116 than to immortalized normal human hepatocyte (MIHA) cells, which may indicate their selectivity. Numerous tests showed the ability of complexes to bind to intracellular proteins such as mitochondrial heat shock protein 60 (HSP60), vimentin (VIM), nucleoside diphosphate kinase A (NDKA), nucleophosmin (NPM), nuclease-sensitive element binding protein (Y box binding protein, YB-1), and peroxiredoxin 1 (PRDX1). The fact that the complexes have multiple molecular targets can minimize the occurrence of drug resistance that is usually encountered with single-target anticancer agents due to naturally occurring genetic mutations [123].

### 2.4. Chlorite–Cyanide Complex of Gold (III)

Recently we characterized the synthesis, in vitro safety as well as anticancer activity of a novel chlorite–cyanide complex of gold (III) named TGS121 (patent no: PL422125A1). The compound was prepared as described by Krajewska et al. The obtained Au(III) complex with the formula [Au(CN)_4_]_2_ (ClO_2_)Na is a sodium salt of chloride dioxide associated with Au(III)–cyanide group complex. This complex is water soluble, stable in a neutral pH and can be kept at room temperature. This complex turned out to be stable in cell culture medium and serum. Its relatively low molecular mass (692.5 g/mol) and the form of sodium salt makes it stable in physiological fluids and enables the passage through biological membranes such as the cell membrane. The novel Au(III) compound turned out to exert cytostatic/cytotoxic effects in cancer Ha-Ras transfected NIH3T3 fibroblasts selectively in comparison to the noncancer NIH3T3 cells (Table 5). Since Ras isoforms share 80% identity and share similar activity—one can conclude that in CRC with Ras hyperactivation (mostly K-Ras) (like HCT116), the compound TGS121 would also be effective [124,125].

## 3. In Vivo Studies

Promising cytotoxicity of many Au(III) complexes has been observed against tumor cells in vitro however, to our knowledge, there has been very little evaluation of these compounds on in vivo tumor models.

Thiosalicylate derivative of cycloaurated Au(III) **4** was tested in vivo against the colon HT29 tumor xenograft. Its cytotoxicity did not translate into in vivo pharmacological activity, with only modest inhibition of tumor growth. The lack of activity could in part be explained by the poor solubility of this complex, indicating that further work is necessary to improve both solubility and lipophilicity in order to improve biodistribution [100].

The second compound tested in vivo was an organogold(III) complex **26** (Figure 10), which exhibits promising in vitro cytotoxicity, but it failed to inhibit in vivo tumor growth in HT29 colon cancer xenografts [126].

Only two described Au(III) compounds exhibited favorable antitumor properties both in vitro and in vivo. The tetraarylporphyrin Au(III) complex **17**, after intraperitoneal injection in mice at doses of 1.5 mg/kg and 3.0 mg/kg, significantly inhibited the proliferation of Colo205 tumor cells, induced apoptosis and inhibited colon cancer tumor growth [114]. The second one, Au(III) mesoporphyrin IX dimethyl ester **19**, after intravenous injection twice per week for 21 days in nude mice bearing human colon cancer HCT-116 at doses 2 mg/kg, resulted in suppression of tumor growth by 72% compared to mice treated with vehicle control [93]. Acute toxicity studies have confirmed that the test compounds, in therapeutic concentrations, do not exhibit additional side effects in mice. Compound **19**, after being transiently present in the liver and kidneys, was completely excreted in the urine. In contrast, complex **17** accumulated in the liver and kidneys with low urinary excretion [93]. The presented results suggest that these complexes may be a new potential therapeutic drug for colorectal cancer.

## 4. Scope and Limitations

This article is limited to gold compounds acting on human CRC cell lines, which might poorly represent the clinical disease. However, especially in CRC surgical treatment, neoadjuvant and adjuvant therapy is applied to reduce the micrometastatic area, and in this context, cell-based assays, especially clonogene assays, can mimic the in vivo situation [35]. We are observing a significant increase in publications on gold complexes. This indicates a great interest in this subject. Review work is also needed to systematize and summarize knowledge about this group of compounds.

## 5. Conclusions

Metallodrugs are very promising due to the fact that, depending on the choice of metal, its oxidation state, type, and number of coordinated ligands, we obtain a unique mechanism of drug action [72]. Therapies based on Au(III) compounds are particularly interesting and are currently being intensively developed due to their structural similarity to Pt(II) [28].

Colorectal cancer was shown to arise as a result of multiple genetic alternations in both oncogenes and tumor suppressor genes. If the initiated colon epithelium cells bearing mutations in APC or k-Ras can still control the DNA repair due to presence of active and functional p53, we talk about early and late adenoma—that is, nonmalignant and not-yet invasive. However, after genetic alternations that render the p53 pathway inactive, the initiated cells continue their unhampered divisions, despite DNA errors, and become malignant colon cancer cells [8]. That is why in colon cancer therapy, metal-based chemotherapeutics such as platin derivatives are used. Such drugs interfere with DNA, initiating cell-division catastrophe and subsequently cancer-cell death. The effectiveness of established metallodrugs is not always good due to resistance development in genetically instable cancer cells. In this context, novel drugs are needed.

The efficacy of Au(III) in the treatment of CRC has been proven in many in vitro assays using various colon cancer cell lines such as HT29, HT-116, COLO 205, and many others. Au(III) complexes are characterized by high efficiency, selectivity towards cancer cells, and compared to cisplatin display reduced toxicity, a broader spectrum of activity and the ability to overcome tumor resistance [57].

Given the structural and electronic similarity of Au(III) complexes to cisplatin and Pt-related anticancer drugs, it was assumed that their mechanism of action involves binding to DNA. However, it appears that DNA may not be the primary biological target of Au(III) complexes due to studies reporting low DNA binding affinity [115]. Although TrxR inhibition was found to be the main pathway of potent antitumor activity of many Au-NHC complexes [127], the reviewed studies on Au(III)–NHC complexes do not support this mode of action. Notwithstanding the fact that we observe a great deal of scientific interest in Au compounds as drug candidates, we still have insufficient data to describe modes of action and the mechanisms they involve [128].

So far, only two Au(III) derivatives are characterized by high cytotoxicity for colon cancer cells, confirmed by both in vitro and in vivo assays. More advances in metallodrug studies are expected to improve the therapeutic potential of Au(III) in colorectal cancer treatment.

## Figures and Tables

**Table 1:** Comparison of IC_50_ (µM) for complexes **2a**–**2e** and cisplatin (**1**) against selected human cell lines [98].

Symbol	Cell Line					
	SW620 (Colon)	SW1116 (Colon)	HT29/219 (Rectum)	ZR-75-1 (Breast)	HT-1376 (Bladder)	SK-OV-3 (Ovary)
**1**	167	163	17	27	23	23
**2a**	124	119	55	34	30	45
**2b**	51	47	25	45	6,7	20
**2c**	281	238	67	41	13	13
**2d**	205	215	19	36	10	10
**2e**	67	80	36	27	11	11

**Table 2:** Characterization of the anticancer properties of new organogold compounds.

Symbol	Proposed Mechanism of Action	Cell Line	IC_50_ Range (µM)
**3a**–**d**	Bind to DNA	HCT8	8.0 ± 2.5 (**3a**) 11.6 ± 2.0 (**3b**) 29 (**3c**) 28.5 (**3d**) 3.9 ± 0.6 (cisplatin)
**4**	Inhibition of cathepsins B and K	DLD-1 HCT-116 HT-29	3.5 (DLD-1) 5.7 (HCT-116) 11.7 (HT-29)
**5a,b**	Undetermined	Caco-2 HT-29	>120 (**5a,b**) 39.8 (**5a**) >120 (**5b**)
**6**	Intercalation of DNA, inhibition of topoisomerase I, II	SW620	15
**7**	Undetermined	LS-174T	74.0
**8a**–**d**	Undetermined	HT-29	5.2 ± 0.4 (**8a**) 18.1 ± 0.6 (**8b**) 17.7 ± 0.4 (**8c**) 33.7 ± 2.2 (**8d**)
**8e,g**	Induction of apoptosis, G0/G1 cell cycle arrest	HCT-116	47.0 ± 3.1 (**8e**) 67.0 ± 4.8 (**8f**) 14.9 ± 0.6 (**8g**)
**9a**–**d**	Undetermined	LoVo	(2.40 ± 0.04) × 10^−2^ (**9a**) 3.8 ± 0.1 (**9b**) 7.6 ± 0.2 (**9c**) 7.9 ± 0.1 (**9d**)
**10a,b**	Induction of ROS-dependent opening of the PTP.	HCT-116	15.8 ±2.1 (**10a**) 43.6 ± 5.4 (**10b**)
**11**	Modification of MtMP, release of cytochrome C to the cytoplasm, caspase-3 activation; inhibition of proteasome.	Caco-2/TC7	1.00 ± 0.06 (11) 45.6 ± 8.08 (cisplatin) 2.1 ± 0.4 (auranorfin)
**12**	Distortion of DNA double helix	HCT-116	
**13**	Inhibition of the zinc-finger protein PARP-1	HCT116 p53+/+ HCT116 p53−/−	2.1 ± 0.7 14.0 ± 1.1
**14a,b**	14a proapoptotic activation, 14b pronecrotic actions	HCT116 HCT116p53−/−	0.48 ± 0.57 (**14a**) 0.33 ± 0.14 (**14b**) 0.23 ± 0.20 (**14a**) 0.27 ± 0.12 (**14b**)
**15**	Molecular target: sulfur-containing proteins	WiDr	9.8 ± 1.2
**16**	Undetermined	HCT116	>50

**Table 3:** Characterization of the anticancer properties of porphyrin complexes.

Symbol	Proposed Mechanism of Action	Cell Line	IC_50_ Range (µM)
**17a**–**e**	Inducing apoptosis by a mitochondrial death pathway	SW1116 Colo 205 CRL-238 CCL-2134 HCT-15 HCT-15A2	0.20 ± 0.02 0.27 ± 0.02 1.41 ± 0.20 0.65 ± 0.13 0.86 ± 0.15 3.43 ± 0.46
**18a,b**	Inducing apoptosis by intrinsic pathway	HT-29 HCT-116	17.0 (**18a**) 3.5 (**18b**) 16.0 (**18a**) 3.0 (**18b**)
**19**	Inhibition the Trx, peroxiredoxin and deubiquitinases	HCT-116 NCM460	0.06 ± 0.01 1.5 ± 0.15

**Table 4:** Characterization of the anticancer properties of N-heterocyclic carbenes (NHCs).

Symbol	Proposed Mechanism of Action	Cell Line	IC_50_ Range (µM)
**20**	Undetermined	HT-29	12.7 ± 1.2
**21**	Undetermined	HCT-116	5.9 ± 3.6
**22**	Undetermined	HCT-116	6.78 ± 2.01
**23**	Undetermined	HCT-116	21.25 ± 1.37
**24a,b**	Inhibition of TrxR	HT-29	6.2 ± 1.0 (**24a**) 7.5 ± 2.9 (**24b**)
**24c,d**	Undetermined	HT-29	0.26 ± 0.03 (**24c**) 0.30 ± 0.01 (**24d**)
**25a**–**g**	Multiple molecular targets	HCT-116	4.40 ± 1.50 (**25a**) 1.10 ± 0.28 (**25b**) 0.49 ± 0.12 (**25c**) 0.23 ± 0.11 (**25d**) 0.25 ± 0.10 (**25e**) 0.52 ± 0.34 (**25f**) 0.20 ± 0.06 (**25g**)

**Table 5:** Characterization of the anticancer properties of TGS121.

Symbol	Proposed Mechanism of Action	Cell line	IC_50_ Range (µM)
TGS121	Apoptosis induction, inhibition of Ras-mediated pathway, cell cycle arrest at G2/M	Ras-3T3	0.231 ± 1.2
		NIH3T3	5.05 ± 2.6

## Data Availability

Data are contained within the article.
